# Harmonizing the interpretation of genetic variants across the world: the Malaysian experience

**DOI:** 10.1186/s13104-015-1798-0

**Published:** 2016-02-26

**Authors:** Nik Norliza Nik Hassan, John-Paul Plazzer, Timothy D. Smith, Hashim Halim-Fikri, Finlay Macrae, A. Zubaidi AL, Bin Alwi Zilfalil, Members of Malaysian Node of HVP

**Affiliations:** Department of Colorectal Medicine and Genetics, The Royal Melbourne Hospital, Victoria, Australia; Human Variome Project, The University of Melbourne, Victoria, Australia; Faculty of Medicine, Medical Campus, Universiti Sultan Zainal Abidin (UniSZA), Jln. Sultan Mahmud, 20400 Kuala Terengganu, Terengganu Malaysia; Department of Pediatric, School of Medical Sciences, Health Campus, Universiti Sains Malaysia, Kubang Kerian, 16150 Kota Bharu, Kelantan Malaysia; Department of Medicine, The University of Melbourne, Victoria, Australia

**Keywords:** Sharing genetic data, Variant database, Variation nomenclature

## Abstract

**Background:**

Databases for gene variants are very useful for sharing genetic data and to facilitate the understanding of the genetic basis of diseases. This report summarises the issues surrounding the development of the Malaysian Human Variome Project Country Node. The focus is on human germline variants. Somatic variants, mitochondrial variants and other types of genetic variation have corresponding databases which are not covered here, as they have specific issues that do not necessarily apply to germline variations.

**Results:**

The ethical, legal, social issues, intellectual property, ownership of the data, information technology implementation, and efforts to improve the standards and systems used in data sharing are discussed.

**Conclusion:**

An overarching framework such as provided by the Human Variome Project to co-ordinate activities is invaluable. Country Nodes, such as MyHVP, enable human gene variation associated with human diseases to be collected, stored and shared by all disciplines (clinicians, molecular biologists, pathologists, bioinformaticians) for a consistent interpretation of genetic variants locally and across the world.

## Background

The management of inherited diseases are a major problem in the health care system. In addition to the complexity and heterogeneity of the clinical data, patients who receive treatment from multiple care centres also cause the fragmentation of important information regarding the diseases. The creation of databases that allow comprehensive access to basic clinical and genetic data will greatly improve accurate diagnosis and treatment of patients with genetic disorders.

The Human Variome Project is an international non-governmental organisation working to build capacity in the practice of responsible genomics. To ensure that this contributes to improving global health outcomes, The Human Variome Project focusses on increasing both the quality and quantity of genomic knowledge that is collected, curated, interpreted and shared for clinical practice. The Human Variome Project acts as an umbrella organisation across multiple countries, institutions and initiatives to establish collaboration around its central vision—the responsible, free and open online publishing of variants and associated clinical data and other information to assist interpretation of variants.

The systematic collection of human genetic variation is an ambitious goal with vast technical and organizational challenges, which must take into account the differing ethical, legal and social norms that exist across the world. The Human Variome Project proposes two main mechanisms to meet the challenges of free and responsible sharing of genetic variant information pertaining to human disease. One is to assist countries in developing their own capacity to generate and contribute their own variant information via the establishment of HVP Country Nodes. The other is to support international groups formed around specific genes or diseases to do the same [1].

HVP Country Nodes are a vital component for achieving improved health outcomes for people both within the country and around the world. An HVP Country Node is defined as having three components: (1) A central repository or network of databases, that receive and store information on variation in the human genome that has been generated within each country to enable the sharing of that information both nationally and internationally; (2) a governance structure that ensures that the work of the Node is both sustainable in the long term and is consistent with all relevant national and international ethical, legal and social requirements; and (3) a set of policies and procedures that ensures that the repository is operated and maintained in a responsible and accountable manner that is consistent with both national and HVP standards.

## Gene/disease-specific variant databases

One of the advantages of an HVP Country Node is the role it can play in supporting gene-disease specific databases. For example, the International Society for Gastrointestinal Hereditary Tumours (InSiGHT) is a professional body of healthcare workers dedicated to advancing knowledge and management of familial gastrointestinal (GI) cancer. Its mission includes the facilitation of international collaborative projects among its members, to support databases of variants related to familial gastrointestinal tumours, and to apply its robust system to assign pathogenicity status to gene variants. The InSiGHT DNA Mismatch Repair (MMR) database [2] forms a primary store of public information of inherited gastrointestinal cancer gene variants and uses Leiden Open Variation Database (LOVD http://www.lovd.nl/) to consolidate data from various sources such as published literature, diagnostic laboratory submissions, clinical reports, research data, and other databases [3]. DNA sequencing techniques have improved leading to an expanding amount of published reports relating to MMR genes. The shared interests between InSiGHT and the Human Variome Project have driven a substantial increase in submissions of MMR variants to the central database. Major outcome of the pilot project initiated by InSiGHT and the Human Variome Project was the establishment of Gene/Disease Specific Databases (GDSDb) as an integral part of the Human Variome Project vision. The formation of GDSDb is to allow gene/disease specialists to value-add to the data collected in Country Nodes such as providing expert interpretation of variant effects.

## National databases

A number of countries have established nationwide efforts to collect genetic variant data. The Australian Node of the Human Variome Project was the first official HVP Country Node, established in Australia in 2009. Funded by the Australian federal government, the project played a role in installing and testing a data collection system in diagnostic laboratories. The initial phase was completed in mid-2011 and the data was made accessible to researchers, clinicians and diagnostic labs throughout the country. The Australian Node does not currently share data internationally. In China, by using the LOVD platform, the Centre for Genetic and Genomic Medicine has begun their data collection in collaboration with healthcare industry, basic research organisations, and industry partners. In the United Kingdom, using proprietary software, the Diagnostic Mutation Database (DMuDb http://www.ngrl.org.uk/Manchester/dmudb.html) was formed to collect data directly from diagnostic laboratories. A Consortium of Arab Genetics Societies (CAGS) developed a database of disease diagnosed in Arab populations [4]. In France, the Universal Mutation Database (UMD http://www.umd.be) has served as a central store for genetic data produced in a network of French laboratories. The UMD software is tailored specifically for a variety of genes associated with cancer and genetic diseases [5].

Endeavours to centralise genetic variants does not necessarily follow national boundaries or disease priorities. For example, the Faroe Islands, with a population of 50,000 has planned on sequencing the entire population [6]. This will expand the focus from a small number of highly prevalent diseases in the Faroes, to a more comprehensive approach that will bring personalised medicine based on each individual’s genetic sequence.

## Genome-wide variant databases

The other main type of variant database are large centralised collections of variants from all genes in the genome. One of the earliest and most well-known is dbSNP (http://www.ncbi.nlm.nih.gov/SNP), which collates variants from humans and other organisms [7]. It’s collection of variants is not limited by disease-association or other criteria, so it is a clearing-house for all types of variation. ClinVar is a recent initiative of the National Center for Biotechnology Information (NCBI) to centralise variants from submitting organisations, particularly information from diagnostic laboratories. It aims to display the assertions regarding the variant’s impact on phenotype, and provides free access to the data. The Human Gene Mutation Database (HGMD) collates genetic variants from published literature. It is available to registered users in free and commercial versions—with the paid subscription providing the most updated data [8].

## Initiating an HVP country node: the Malaysian experience

The Malaysian Human Variome Project (MyHVP) (http://hvpmalaysia.kk.usm.my/) was launched on the 9th October 2010. To date, 71 individuals from 12 Malaysian universities and academic institutions form the membership of MyHVP. MyHVP has succeeded in developing an online portal for the mutation of genes related to genetic diseases prevalent in Malaysia and also the Malay whole genome SNP data. MyHVP marks a new horizon for genetic activities in Malaysia and provides a database on genetic variation that relates to the Malaysian population. The most recent development is the establishment of the Southeast Asia (SEA) regional node of the Human Variome Project (HVP SEA Node), which is expected to bring together individuals working in diagnostics and clinical care within Malaysia and Southeast Asia (SEA). The HVP SEA Node was established through Malaysia’s position as the regional role model in genomic research and diagnostic services especially for Southeast Asian developing countries. It is believed that the formation of MyHVP will be able to assist these countries by providing training and education in order to increase public awareness on the importance of genetics and genomics in health care. By emphasizing the four areas of activities for the Human Variome Project which are setting normative functions, behaving ethically, sharing knowledge and building capacity, MyHVP will ultimately be a referral center for SEA variome activities. This will elevate the status, enhance the visibility and highlight the contributions of Malaysia in the field of genetics, genomics and variomics.

## Administrative activities of a country node

There are diverse issues that needed to be considered in the formation of HVP Country Node: medical and genetic information privacy, Information Technology, Ethical/Legal/Social Issues, (ELSI), and organisational structure. A wide variety of expertise and co-operation between different groups is required to successfully implement a country node. The critical component in the initiation and development of the Country Node is a local well-informed and enthusiastic champion to drive the project forward. Most importantly, an HVP Country Node should be formed in association with the respective local human genetics/genomics society. Therefore, the formation of MyHVP received full support not only from the highest administration level of the university, but also from local societies such as Genetics Society of Malaysia, the Malaysian Society of Human Genetics, the Medical Genetics Society of Malaysia, and the Malaysian Society of Bioinformatics and Computational Biology. The Malaysian Ministry of Sciences, Technology and Innovation (MOSTI) and Ministry of Education (MOE) supported the project at several government levels including the Malaysian Ambassador to UNESCO. Creating an HVP Country Node also requires support from diverse stakeholders, from patient advocacy groups through local and national government agencies. MyHVP received the funding from Universiti Sains Malaysia through APEX Grant (1002/PPSP/910343) with the amount RM790,959.32 since November 2012 to set up the MyHVP Country Node. This amount is for advocacy of MyHVP and genomic research such as the sequencing project for the Malay ethnicities. At the administrative level, support from clinicians, diagnostic personnel, geneticists, bioinformaticians and other important stakeholders within the country has helped in initiating and sustaining the project.

## The ICT infrastructure system

The Malaysian Node of the Human Variome Project (MyHVP) Database used open source software in the development of the variation and mutation databases. The database was created to organise, store and distribute variations and mutations that are related to population specific Malaysian ethnic groups. The following freely available open source software uses the PHP scripting language v.4.2.0 and up (http://www.php.net), and MySQL database package v.3.23.33 and up (http://www.mysql.com). The MyHVP Database has been constructed based on a three-tier architecture model i.e. client, web and database. PHP scripts are used as a common gateway interface (CGI) for sending and receiving data between the front end user/client and the database server. To query data, users need to enter or choose a keyword such as SNP ID or chromosome number or Gene ID. The Apache web server then transforms the query into SQL for it to be requested from MySQL database.

In addition, MyHVP is planning to implement an LOVD system, which is also written in PHP and uses MySQL open source software. It was developed to allow the easy creation and maintenance of a gene sequence variation database using the internet. The gene-centred design of the database follows the recommendations of the Human Genome Variation Society (HGVS http://www.hgvs.org/) and focuses on ease of use and flexibility. The newest LOVD version 3, released late 2012, also allows storage of Massively-Parallel Sequencing data, which often results in large numbers of variants found in between genes as well. To ensure the use of unambiguous sequence variant descriptions in newly submitted data, LOVD interacts with Mutalyzer [9] to apply the HGVS human nomenclature guidelines to check and enforce correct sequence variant descriptions. The usual focus of LOVD is the connection between a gene and a heritable disease. Sequence variants found in individuals are imported or submitted into the database, together with information about whether they could be causally related to the disease. Specialized doctors (clinical geneticists) can use LOVD disease specific databases to assist in interpreting diagnostic sequencing data and advise patients confirmed as carrying a genetic disease. Ideally, if a patient has been screened for mutations and one has been found, information in LOVD can assist in the diagnosis and progress of this disease. By typing genesymbol.lovd.nl onto one’s browser, the user will be connected directly to the web browser and LOVD database displaying a list of choices for the corresponding gene.

## Ethical/legal/social issues (ELSI)

An important aspect of the development of a Country Node is the multicultural, ethical and legal issues that might contribute to problems in submitting the genetic data. MyHVP will endeavour to resolve these questions over time as they develop in the Malaysian context. Among the issues analysed is whether one has a right to know or a right not to know genetic information. This is in line with the UNESCO Universal Declaration on the Human Genome and Human Rights which states in Article 5c the right of every individual to choose whether or not to be informed of the results and consequences. Currently, there exists no regulatory framework that control genetic testing, screening and gene therapy in Malaysia.

Obtaining consent from human subjects and patients is crucial to any medical testing and research. A survey of research institutes in Malaysia has revealed that there is no standard form and practice in obtaining consent from human subjects. Yet obtaining effective consent from human subjects is key defence to any possible allegation of medical malpractice.

Malaysia’s population comprises many ethnic groups, whereby the total population is 28.3 million, with the Malays comprised 63 % of the total population, followed by Chinese (28 %), Indians (8 %), and other ethnic groups (1 %) [10]. Most of them are actually descended from Javanese, Bugis, and Minang sailors who came from Indonesia, during the 17th to early 20th centuries. The vast majority of ethnic Malaysian Chinese came from the Fujian and Guangdong provinces in Southern China. Malaysian Indians are a group of Malaysians largely descended from those who migrated from southern India during the British colonization of Malaya.

The wide genetic variation produced from the multiracial population will enable researchers to conduct comparative studies between matched cohorts with and without disease. Discovering these variations will provide fundamental new insight into the pathogenesis, diagnosis and treatment of human diseases and towards the development of personalized medicine. Thus, besides being responsible for the funding, collection and data storage, each Country Node needs to ensure the handling of the data is according to the nation’s own law in an ethical and culturally sensitive manner. To alleviate patients’ privacy issues in relation to the database records, the curator needs to have clear guidelines to solve the challenges. The ethical issues in the data-basing of mutations have been discussed seriously especially in the Islamic perspective and recent attempts have been made to tailor some important guidelines from UNESCO, HUGO and WHO that can fit the local curating of phenotype/genotype data [11, 12].

Another issue that surrounds the use of genetic data is when genetic information is taken from corpses. The issue that arises would be the prior informed consent of the family concerned which gives rise to the question of ownership of the corpse. This is an active area for research in terms of genealogy and paternity testing but also in the genetics of diseases. At this juncture, it is important to look into the Islamic position which places due respect on the dead and the rights owed to them and a consideration will need to be made as to whether the collection of genetic information from dead bodies is against the Islamic Law.

## Medical/genetic confidentiality

Collection of DNA samples from live donors implicates confidentiality issues. The medical data is considered to be sensitive data and has raised an argument that any form of DNA sampling and collection would also have to comply with the personal data principles. Therefore, the handling of such data requires extreme care and explore other duties of confidentiality that may arise from common law and other statutory instruments such as Medical Act 1971, Private Healthcare Facilities and Services Act 1998, Child Act 2001. What other usage could a researcher/doctor make of medical/genetic data without offending the data privacy principles? On a broader scale, the collection of DNA samples could also implicate privacy issues. With the enactment of the Personal Data Protection Act 2010 [13], there is a wide exception given for research. However, would the possible aim of developing commercial applications to the research output exclude these activities from the research exception? This is not made clear in the Act.

## MyHVP data collection results

**Fig. 1:**
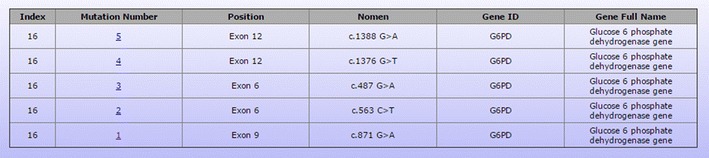
Data structures that were recorded in the MyHVPDb

In the MyHVP Database, the Malay ethnic group has been the first to have available online the 291,718 SNPs dataset which was obtained by genotyping the SNPs of 103 healthy individuals from Champa, Kelantan, Bugis, Banjar, Kedah and Jawa subgroups of Malays. The mutation database also includes 143 mutations from 16 genes related to diseases common in the Malay ethnic group. However, the number of mutations will be increasing as there are on-going studies on disease causing variants. In fact, MyHVP database has curators who are responsible to add new mutations monthly. The current data on these mutations was collected from results published in journals [14]. All mutational data in the MyHVP database were recorded based on the Human Genome Variation Society (HGVS) nomenclature (http://www.hgvs.org/mutnomen/standards.html) (Fig. 1). Detailed description of sequence variants is at the Table 1.

**Table 1:** Sequence variants according to the Human Genome Variation Society(HGVS) (http://www.hgvs.org/mutnomen/standards.html)

Variants	Description
DNA/RNA	Conversion = a sequence change where a range of nucleotides are replaced by a sequence from Elsewhere in the genome Deletion = a sequence change where one or more nucleotides are removed (deleted) Deletion/insertion (indel) = a sequence change where one or more nucleotides are replaced by one or More other nucleotides Note: when one nucleotide is replaced by one other nucleotide the change is called a substitution Duplication = a sequence change where a copy of one or more nucleotides are inserted directly 3’- flanking of the original copy Note: when the copied sequence is not inserted directly 3’-flanking of the original copy the change is called an insertion Insertion = a sequence change where one or more nucleotides are inserted between two nucleotides but where the insertion is not a copy of a sequence immediately 5’-flanking (see duplication) Inversion = a sequence change where more than one nucleotide replacing the original sequence are the reverse complement of the original sequence Substitution = a sequence change where one nucleotide is replaced by one other nucleotide Note: a sequence change where one nucleotide is replaced by more than one other nucleotide is a deletion-insertion (indel) Note: a sequence change where more than one nucleotide is replaced by one or more other nucleotide is a deletion-insertion (indel)
Characters used:	(1) Reference sequences c. = coding DNA reference sequence (2) Numbering I. Genomic, mitochondrial, non-coding RNA, RNA and protein reference sequence N = nucleotide N in reference sequence (e.g. 311A > G) II. coding DNA reference sequence N = nucleotide N in protein coding sequence (e.g. 11A > G) N + M = nucleotide M in the intron after (3′ of) position N in the coding DNA reference sequence (e.g. 30 + 4A > G) 3) Specific characters + (plus) − (minus) > (greater than) = changes to (substitution) c.5T > G substitution 4) Others chr = chromosome (e.g. chr19 or chrX) del = deletion dup = duplication ext = extension (e.g. N- or C-terminus of protein) ins = insertion inv = inversion con = (gene) conversion fs = frame shift t = translocation

## Summary

Collaboration with national and international experts will facilitate the free and open sharing of annotated and curated information. By following the experiences of InSiGHT, the Australian Node of the Human Variome Project and other databases, new processes and systems are under development at MyHVP which are adapted to Malaysian legal and ethical frameworks. A new version of LOVD known as LOVD v3 which is available online will enable more complex data submissions. With the increasing number of data entries in future, the database user interface will be further developed to allow for much easier navigation and customized display of database query results. It is hoped that this article will encourage the systematic integration of variant submission in normal laboratory practice.

## Abbreviations

InSiGHT: International Society for Gastrointestinal Hereditary
MyHVP: Malaysian Node of the Human Variome Project
HVP: Human Variome Project
GI: gastrointestinal
LOVD: Leiden Open Variation Database
GDSDb: gene/disease specific databases
UMD: Universal Mutation Database
LSDB: locus-specific database
DMuDb: diagnostic mutation database
CAGS: Consortium of Arab Genetics Societies
HVP SEA Node: Southeast Asia(SEA) regional node of HVP
MOSTI: Malaysian Ministry of Sciences, Technology and Innovation
MOE: Ministry of Education
UNESCO: The United Nations Educational, Scientific and Cultural Organization
NCBI: National Center for Biotechnology Information, (UCSC Genome Browser): University of California, Santa Cruz
PHP: hypertext pre-processor
SQL: structured query language
HGVS: Human Genome Variation Society
CGI: common gateway interface
SNP: single nucleotide polymorphism
HUGO: Human Genome Organization
WHO: World Health Organization
ART: Assisted Reproductive Technology
ELSI: ethical, legal and social issues
DNA: deoxyribonucleic acid
